# Severity-Based Adaptation with Limited Data for ASR to Aid Dysarthric Speakers

**DOI:** 10.1371/journal.pone.0086285

**Published:** 2014-01-23

**Authors:** Mumtaz Begum Mustafa, Siti Salwah Salim, Noraini Mohamed, Bassam Al-Qatab, Chng Eng Siong

**Affiliations:** 1 Faculty of Computer Science and Information Technology, University of Malaya, Kuala Lumpur, Malaysia; 2 School of Computer Engineering, Nanyang Technological University, Singapore, Singapore; UNLV, United States of America

## Abstract

Automatic speech recognition (ASR) is currently used in many assistive technologies, such as helping individuals with speech impairment in their communication ability. One challenge in ASR for speech-impaired individuals is the difficulty in obtaining a good speech database of impaired speakers for building an effective speech acoustic model. Because there are very few existing databases of impaired speech, which are also limited in size, the obvious solution to build a speech acoustic model of impaired speech is by employing adaptation techniques. However, issues that have not been addressed in existing studies in the area of adaptation for speech impairment are as follows: (1) identifying the most effective adaptation technique for impaired speech; and (2) the use of suitable source models to build an effective impaired-speech acoustic model. This research investigates the above-mentioned two issues on dysarthria, a type of speech impairment affecting millions of people. We applied both unimpaired and impaired speech as the source model with well-known adaptation techniques like the maximum likelihood linear regression (MLLR) and the constrained-MLLR(C-MLLR). The recognition accuracy of each impaired speech acoustic model is measured in terms of word error rate (WER), with further assessments, including phoneme insertion, substitution and deletion rates. Unimpaired speech when combined with limited high-quality speech-impaired data improves performance of ASR systems in recognising severely impaired dysarthric speech. The C-MLLR adaptation technique was also found to be better than MLLR in recognising mildly and moderately impaired speech based on the statistical analysis of the WER. It was found that phoneme substitution was the biggest contributing factor in WER in dysarthric speech for all levels of severity. The results show that the speech acoustic models derived from suitable adaptation techniques improve the performance of ASR systems in recognising impaired speech with limited adaptation data.

## Introduction

Speech is second nature for most of us, to the extent that we cannot imagine how life would be like without it, as speech communication is a vital skill in our society. Inability to communicate verbally is a serious disability that can drastically affect a person's life. Speech impairment deprives a person of communicating with others, and severe speech impairment can be frustrating for both sufferers and listeners.

Several studies show that about 60% of individuals with speech impairments have difficulties in communicating orally with others; such disability severely affects their social life [1]. Speech impairment is sometimes, but not always, the result of cognitive impairment. Thus, some sufferers can learn and make sound judgments, but, due to their poor speaking ability, they have difficulties in communicating with others; this condition affects their ability in learning and restricts their chances of gaining a proper education.

Dysarthria is one of the common types of speech impairments in several studies on ASR systems for impaired speech. It is a motor speech impairment caused by neurological diseases such as cerebral palsy, neurological injuries such as stroke or various other traumatic brain or nerve injuries [2]. Dysarthric speech is characterised by weakness, paralysis or poor coordination of the muscles responsible for speech [3]. As a result, the speech has poor articulation, low precision, and badly pronounced phonemes; it is spoken at a very slow rate and has variable intensity. It is difficult for human listeners to understand this defective speech [4]. On top of that, dysarthria itself is often a symptom of a gross-motor disorder, whose other symptoms often hinder a sufferer from using a keyboard and mouse. Published case studies have shown that some dysarthric users may find it easier to use speech technology such as automatic speech recognition (ASR) systems [5]–[7], instead of a keyboard.

The traditional approach to alleviate the problem faced by speech-impaired individuals is to improve their speaking skill with the assistance of a speech therapist [8]. However, engaging a personal speech therapist is an expensive solution that may not be affordable to most sufferers, where the improvement in speaking skill requires a long period of time.

Recently, speech technology such as automatic speech recognition (ASR) system has offered an alternative solution for individuals with speech impairment to improve their ability to communicate orally. Although ASR was originally intended for enabling oral communication between man and machine, it has been increasingly adopted as an assistive tool for individuals suffering from speech impairments.

An ASR tool is a system where a machine such as a computer is programmed to recognise and act on spoken language. The most familiar usage of ASR systems is the conversion of speech input into text output. ASR systems use several components to recognise speech, one of the most important being the acoustic model. An acoustic model is created by taking audio recordings of speech and their text transcripts, and using software to create a statistical knowledge base of the sounds that make up each word in a process known as speech training.

The issue of building a high-quality acoustic model is arguably the most complex and the most important one in ASR systems [9]–[11]. This is because the acoustic model depends on both the quality and quantity of the recordings. However, in ASR system development, one prominent issue in acoustic model building is the availability of a speech database with sufficient quality and quantity. The lack of speech database is more apparent for the speech-impaired community, where their physical limitation hinders the accumulation of sufficient data to fill a speech database large enough for building an effective speech acoustic model [12]–[14].

Due to the scarcity of speech databases available for dysarthric speakers, the creation of an acoustic model trained exclusively in pathological speech is a difficult task. As such, two alternatives can be considered when developing ASR systems for people with dysarthria. The first one, which is a crude approach, is to use an unimpaired (normal speech) speech acoustic model to recognise impaired (dysarthric) speech. However, the speech of a dysarthric speaker has very low speech intelligibility, causing typical measures of speech acoustics to have values in ranges that are very different from those for unimpaired speech [15]. It is unlikely then that the acoustic models trained in unimpaired speech will be able to adjust to this mismatch [16], [17].

An alternative solution to the scarcity of dysarthric speech is the use of adaptation techniques where a source model, which can be an unimpaired speech acoustic model, is transformed to be more reflective of the speech features of dysarthric speakers. Adaptation seems to be an approach worth pursuing to overcome the obstacles in developing ASR systems for dysarthric speech.

However, some of the issues that are not well addressed in existing research on ASR systems for dysarthric speakers include the use of the most effective adaptation technique and the most suitable source model for performing model adaptation. This is because an acoustic model derived from model adaptation must be able to interpret variations of speech properties for differing levels of severity of dysarthric speech [4], [5], [18].

The remaining sections of the paper are organised as follows. In Section 2, we briefly describe types of existing ASR speech acoustic models and available types of speech acoustic models for dysarthric speakers. Section 3 describes various techniques for adaptation of dysarthric speech and the most suitable source model for adaptation. Section 4 describes the methodology used in this research and includes information on the comprehensive development of different techniques for adaptation, dataset for training the speech acoustic model, performance measurement methods and procedures. Results and discussions are presented in Section 5 and 6 respectively. Finally, Section 7 serves as the conclusion with a summary of the major findings.

### A. ASR Speech Acoustic Models

There are three categories of speech acoustic models for ASR differentiated by the degree of user training required before use, which are speaker dependent, speaker independent and speaker adaptation ASR.

Speaker dependent (SD) acoustic modelling requires speaker training or enrolment before use, and a primary user trains the speech recogniser with samples of his/her own speech. These systems typically work well only for the person who trains it. The recognition accuracy of the SD ASR system is very high when recognising speech of the intended speaker but yields poor results for users who did not perform voice training before using the system [9], [10], [13].

The speaker independent (SI) acoustic model is the exact opposite of the SD model; this form of ASR does not require any speaker training before use. SI ASR system is pre-trained during system development with speech samples from a collection of many different speakers. An ASR system with SI acoustic model can be used to recognise voices of many different speakers with relatively high accuracy if their speech falls within the range of the collected samples. However, the accuracy of a speaker-independent ASR system will generally be lower than that produced by a speaker-dependent ASR system. While an ASR system with SI model can show impressive performance, its recognition error (in terms of word error rate) can be twice or three times greater than that for the SD acoustic model [9], [10].

The speaker adaptation (SA) speech acoustic model is similar to the speaker-independent ASR in that no initial speaker training is required before use, and it has attracted much attention over the last decade. However, unlike SI ASR systems, as the SA ASR system is used, the model gradually adapts to the speech of the user. Hence, the rationale for developing an ASR system that applies the SA model is its ability to recognise speech comparable to that of the SD model, but requires only a small fraction of speaker-specific training data needed to build a full SD system. The SA model is also useful when there is a scarcity of speech samples of intended users such as those suffering from speech impairment [12]–[14], [19].

#### I. Speech Acoustic Model for Dysarthric Speakers

Earlier research has evaluated different types of speech acoustic models and performance of ASR systems in recognising dysarthric speech [1], [12]–[14], [16]. Sanders et al. [17] conducted a comparative study to evaluate the performance of SD and SI models with dysarthric speech. The SI systems were trained with unimpaired speech of 5,000 speaker corpuses (40 items per speaker), whereas the SD systems were trained with the speech of two dysarthric speakers for 8.5 and 12.8 minutes of speech respectively. The SI and SD speech acoustic models were tested using ten dysarthric speakers; each speaker uttered ten utterances. It was found that the performance of the ASR systems using the SD model was better than that of the systems using the SI model. The recognition error of the SI system was between 67–100%, whereas the recognition error of the SD system was 19–50%; this marks an improvement in recognition of 50–100% over that of the SI model. Similar findings were also reported in [15], [19]. However, building SD model for speech-impaired individuals is non-trivial because of difficulties in obtaining sufficient speech data for training from an individual afflicted with dysarthria, particularly at the more severe level [1], [12], [17].

One of the earlier speech databases available for dysarthric speakers is Nemours [20], which has been applied in numerous research projects on ASR for speech impairment [16], [21], [22]. The Nemours database contains the voices of 11 dysarthric speakers, each uttering 74 nonsensical sentences. It also contains the speech of 11 non-dysarthric speakers uttering the same sentence sets for control purposes. However, it is not large enough for building SI model for dysarthric speech.

Recent and larger speech databases such as the TORGO [23] and the UAspeech databases [24] offer researchers the opportunity to build efficient SI and SA ASR systems for dysarthric speech. The TORGO database consists of more than two hours of recordings of dysarthric speakers, which are suitably applied to the SI dysarthric speech acoustic model. However, the UAspeech database, which is based on isolated words (digits), is not a suitable database to develop a continuous speech recognition system.

As mentioned earlier, the speaker adaptation model seems useful for dysarthric speech recognition since dysarthric subjects find it very exhausting to record large amounts of speech for training ASR systems. Several studies have been carried out on the SA model for dysarthric speech [16], [17], [25].These studies have shown that the SA model can be a good alternative to ASR systems, especially for more severely speech-impaired dysarthric speakers with limited or no database. However, the researchers did not consider the adaptation techniques and the source models that can be suitably applied to optimise the performance of ASR systems in recognising dysarthric speech.

Raghavendra et al. [26] compared recognition accuracy of SA ASR system with that of SD ASR system; the authors found that the SA ASR system adapted well to speech of speakers with mild or moderate dysarthria, but the subject with severe dysarthria was able to achieve better performance with the SD system than with the SA system. These findings were also supported by Rudzicz [25] who compared the performance of SD and SA systems using the Nemours database; the researcher independently varied the quantity of data for training and the number of Gaussian components used for modelling the output probability distributions. The SA technique implemented is not a speaker adaptation approach in the conventional sense: it uses parameter values for the SI system as the starting point to train the speech acoustic model for a particular dysarthric speaker.

Recently, Sharma and Hasegawa-Johnson [27] investigated the development of medium vocabulary recognisers for dysarthric speech of various degrees of severity. The most interesting outcome of their study was that for subjects with very severe dysarthria, model adaptation was able to achieve substantial improvement in recognition accuracy compared with the SD systems. This finding is significant in that it is contrary to the conclusions of previously published studies [25], [26]. Although some of the existing studies on adaptation for dysarthric speech did not disclose the adaptation technique used, they can be broadly categorised into two areas, which are MAP and MLLR. These techniques are described below (refer to Section 3).

### B. Adaptation of Dysarthric speech

A good source model is vital for performing SA, as it will directly affect the target speech acoustic model. For ASR systems of impaired speech, some of the source models applied includes unimpaired and impaired speech models (preferably SI model).The effect of the adaptation technique is influenced by the amount of adaptation data that are used to build a target model based on a source model. When adaptation data consist of those of a single speaker, a general SI acoustic model can be transformed into SD acoustic model. When enough adaptation data are available in a new domain for every single speaker, it is possible to create SD acoustic model for every speaker. Unfortunately, there is not always enough adaptation data available to create SD model for every speaker in a new task.

The issue to be addressed is the way to optimise the use of such limited data. Adaptation techniques, notably the maximum likelihood linear regression (MLLR) [28] and the maximum a posteriori (MAP) [29], are used in large vocabulary continuous speech recognition (LVCSR) to tune SI recognisers to the speech of an individual, resulting in SD model, with a relatively small amount of adaptation data. There is, however, an assumption in these procedures that the target speech is not a gross mismatch to that used to train the SI models.

MLLR is a technique typically used for speaker adaptation purposes in speech recognition systems. MLLR [28] adapts the observation probability of the hidden Markov models (HMMs) in a parametric way, where it finds a transform vector that maximises the likelihood of the adaptation data from a set of transformed Gaussian parameters. HMMs are widely used in pattern recognition applications, most notably speech recognition. A HMM consists of a recurrent finite-state Markov chain, and a distribution over that state for each transition in the Markov Chain. The states and transitions of the Markov chain are hidden from observation so that only the output symbols are visible [30]. Transitions between the states are governed by a set of probabilities called transition probabilities, using the following formula:
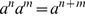
(1)


Where a_ij_ is the state transition probability, q_t_ denotes the current state, and N is the number of states. Unlike the standard MAP adaptation, which adapts only the observed Gaussian components, MLLR transforms are typically estimated across a set of Gaussians, a regression class that shares the same transformation parameters [31]. In the general MLLR framework, both mean and variance parameters are transformed as follows [31]:
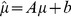
(2)

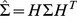
(3)Where μ is a mean vector in the model, Σ is its corresponding covariance matrix, 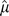 and 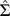 are the adapted mean and covariance matrix, respectively. The likelihood function of the adaptation data are to be maximised with respect to the transform parameters (A;b;H). This is performed using the expectation maximisation (EM) in two steps [28], by estimating the covariance transformation, H after the mean transform of A and b.

The MLLR transformation is applied to all model parameters irrespective of whether they have been observed in the adaptation data. This is achieved by allowing transformation to be shared among the groups of model parameters, by using regression classes. The basic assumption is densities that are assigned to the same transformation class exhibit similar acoustic properties and can therefore be transformed in the similar way. This means that the MLLR technique is suitable for target data that are of small amounts and are incomplete.

The main problem of the MLLR adaptation technique is the reliability of estimating the regression coefficients from the available training data. The constrained MLLR (CMLLR) [32] is an extension of the MLLR technique whereby the former performs transformation of the mean vectors while the latter performs both the mean vectors and the covariance matrix adaptation. CMLLR simplifies the regression model by using diagonal or block-diagonal covariance matrices [28], thereby reducing the number of parameters in the linear regression model, or to share the mean and the variance transforms. For an arbitrary Gaussian component in a regression class, the parameters are transformed using CMLLR as follows [31]:
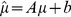
(4)

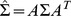
(5)Where the linear transform **A** is used for the adaptation of both μ and Σ. The main difference in CMLLR from MLLR is that, using the same number of parameters, the covariance matrices are also adapted. The algorithm used for the CMLLR adaptation is normally the same as that used for MLLR such as EM. In CMLLR, sufficient statistics are computed for the current estimates, and the likelihood function is maximised with respect to these parameters [31]. CMLLR can be used in speaker-recognition systems to extract features that are more specifically focused on the speaker-related characteristics than the standard spectral envelope features [33].

MAP considers model parameters to be random variables with a known prior distribution [27]. Given a model parameter *θ* with a prior distribution *f (θ)* and sequence of observations *O = {O_1_,O_2_,O_n_}*, the MAP estimate for *θ* is given by:
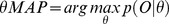
(6)

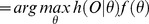
(7)


Where *h (O|θ)* is the density function for observation O, from which the model parameters estimate *θ* is to be estimated. In the case where no knowledge exists regarding the prior distribution *f (θ)*, the MAP estimate equates to the maximum likelihood estimates. In contrast to MLLR, in MAP, only the parameters for which observation exists are adapted. As the amount of adaptation increases, most of the original parameters are adapted and, in principle, the MAP estimates converge towards the maximum likelihood estimates.

MAP and MLLR have been applied in several studies for the adaptation of dysarthric speech. For instance, the MAP technique has been applied in [27], [34], and the MLLR technique has been applied in [19], [34], [35] with acceptable recognition rates. In [36], a comparative study has been conducted to compare the performance of the MAP and MLLR techniques in terms of the recognition of impaired speech; it was found that MLLR performed better compared with MAP. However, no existing work has been carried out on the performance of CMLLR for dysarthric speech.

## Methods

This research experiments with different adaptation techniques and source models that can be suitably applied for the optimum performance of an ASR system in recognising dysarthric speech. An adapted acoustic model for recognising the dysarthric speech of Nemours was built by adapting two SI models based on the unimpaired TIMIT [37] and the impaired TORGO [23] speech; the aim was to assess the suitability of these two SI models as the source model and identify any emerging differences between the models. The performance of the ASR system under each identified adaptation technique (MLLR and C-MLLR) and the source model (unimpaired and impaired speech) is measured in terms of the word error rate (WER) for each level of severity of impaired speech (mild, moderate and severe). We have performed a statistical analysis to determine any significant difference in the variance of WER. This section describes the databases, research methodology, performance measures and equipment involved in our experiments.

### A. Speech Databases

The Nemours database contains the speech samples of 11 dysarthric males and one non-dysarthric male, each uttering 74 syntactically invariant short English sentences and two additional paragraphs [20]. The speakers of the Nemours database have been categorised according to three types of speech impairment severity, which are mild, moderate and severe. These classifications were based on the intelligibility scores in [26], [38]; speakers with intelligibility scores exceeding 80% were classified as mild, whereas speakers with intelligibility scores of less than 60% were classified as severe, and the rest of the speakers were classified as moderate as shown in Table 1. The classification is crucial to determine the performance of the ASR system in recognising the dysarthric speech for the different levels of severity. From the 11 speakers of Nemours, nine speakers were chosen; the speech of speaker KS was omitted because of missing and incomplete records, and the speech of speaker LL was left out to maintain the same number of speakers for each severity type.

**Table 1:** The classification of speakers in Nemours database.

Speakers	Intelligibility score	Severity Types
FB	92.9	Mild
MH	92.1	Mild
BB	89.7	Mild
LL*	84.4	Mild
JF	78.5	Moderate
RL	73.3	Moderate
RK	68.6	Moderate
BK	58.2	Severe
BV	57.5	Severe
SC	51.5	Severe
KS*	Not available	Unknown

* Not included for model adaptation.

The TIMIT Acoustic-Phonetic Continuous Speech Corpus is widely used for both speech and speaker recognition tests, and each utterance is phonetically hand-labelled. TIMIT contains recordings from 630 English speakers (438 males and 192 females). After excluding dialectal variants, a total of 5,040 sentences were used as the training data to build the SI model of unimpaired speech. An experiment conducted in [40] showed that phoneme recognition performed well on the TIMIT speech corpus when the HTK toolkit used the Bigram language model.

TORGO consists of seven dysarthric subjects' speech, each uttering three hours of data (about 500 utterances each). 3,500 sentences were used for the training data to build the SI model of impaired speech.

### B. Research Procedures

The procedure of this research begins with the process of building SI model for TIMIT and TORGO. This is followed by the model adaptation of Nemours for building SA (adapted) speech acoustic model to be applied by an ASR system for recognising impaired speech. The detailed procedure is described below.

#### I. Building Source SI Models

The baseline SI model built with TIMIT and TORGO was developed using the HTK toolkit [39]. A Hamming window of 25 milliseconds moving at a frame rate of 10 milliseconds was applied to the waveform data to convert them to 12 MFCCs (using 26 filterbanks); the energy, delta and acceleration coefficients were then added. The standard three state left-to-right HMM topology was applied during training with the standard maximum-likelihood technique.

#### II. MLLR and C-MLLR Adaptation for Dysarthric Speech

Each of the baseline SI models (TIMIT and TORGO) is adapted using Nemours, which has been categorised into three levels of severity: mild, moderate and severe. All the adaptations (MLLR and CMLLR) make use of a single Gaussian mixture model [31], and we have applied the BiGram statistical language model of the respective speech databases.

The MLLR and CMLLR adaptations of the Nemours database based on the severity levels are described in Table 2, including the source models of TIMIT and TORGO, the severity levels (mild, moderate and severe), the adapted models (mild, moderate and severe) and the test data of Nemours. The adapted model was built using 50 sentences from each dysarthric speaker of Nemours, while the remaining 24 were used for testing.

**Table 2:** The experimental set-up of this study.

Experiment	Source SI model	Severity level	Adapted dysarthric model	Test data
MILD-A	TIMIT	Mild	1. Speaker FB	1. Speaker FB
MILD-B			2. Speaker BB	2. Speaker BB
MILD-C			3. Speaker MH	3. Speaker MH
MOD-A		Moderate	1. Speaker JF	1. Speaker JF
MOD-B			2. Speaker RK	2. Speaker RK
MOD-C			3. Speaker RL	3. Speaker RL
SEV-A		Severe	1. Speaker SC	1. Speaker SC
SEV-B			2. Speaker BK	2. Speaker BK
SEV-C			3. Speaker BV	3. Speaker BV
MILD-A	TORGO	Mild	1. Speaker FB	1. Speaker FB
MILD-B			2. Speaker BB	2. Speaker BB
MILD-C			3. Speaker MH	3. Speaker MH
MOD-A		Moderate	1. Speaker JF	1. Speaker JF
MOD-B			2. Speaker RK	2. Speaker RK
MOD-C			3. Speaker RL	3. Speaker RL
SEV-A		Severe	1. Speaker SC	1. Speaker SC
SEV-B			2. Speaker BK	2. Speaker BK
SEV-C			3. Speaker BV	3. Speaker BV

### C. Performance Measure

We have applied the leave-one-out evaluation for measuring the effectiveness of the two SI models. The effectiveness of an ASR system is generally measured in terms of word error rate (WER). WER measures global and incorrect word recognition in a total recognition task. As an alternative, an error rate may also be measured in smaller units such as phonemes, syllables or detailed errors, including phoneme insertion, substitution and deletion rates [41] as follows:
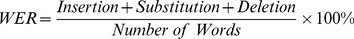
(8)Where:

- *Phoneme insertion:* An extra sound or sounds added to the intended word due to slow speaking rate of a dysarthric speaker, which causes a monosyllabic word to be interpreted as two syllables.
- *Phoneme substitution:* One phoneme is substituted with another due to errors in pronunciation made by people suffering from dysarthria, e.g. twee instead of tree.
- *Phoneme deletion:* Certain sounds are not produced by the people suffering from dysarthria, which causes all the syllables or specific sounds to be omitted.

For example, the sentence “the two weeping the bit” uttered by speaker BB is recognised by the ASR system as “the big is sleeping the bet”. The WER for this test data is calculated as shown in Table 3.

**Table 3:** The WER calculation for the uttered sentences “The two weeping the bit” in terms of phoneme addition, substitution and omission.

	Error		
Input word	Phoneme Insertion	Phoneme Substitution	Phoneme Deletion
The			
two		big	
weeping	sleeping	sleeping	
the			-
bit		bet	

Based on Table 3, the WER of the sentence “the two weeping the bit” can be calculated as follows:
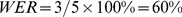
(9)The WER of the sentence can be analysed into addition as 15% (1/4×60%), substitution as 45% (3/4×60%) and omission as 0% (non-occurrence). The sum of all the three errors of addition, substitution and deletion is equal to the WER of the sentences “the two weeping the bit”, which is 60%.

### D. Statistical analysis

We have performed the ANOVA test to measure any significant difference in the WER between the two techniques globally and at each level of severity with TIMIT being the denominator and TORGO as the numerator. The ANOVA test is also performed for the WERs arising from the phoneme insertion, substitution and deletion for the different levels of severity.

## Results

The recognition of dysarthric speech using the TIMIT SI model is more accurate for the mildly impaired speech (for both adaptation techniques), while the one based on TORGO performs well in recognising the moderately impaired and the severely impaired speech (for both adaptation techniques) except for experiment MOD-A. The CMLLR technique shows a lower WER than the MLLR technique for both the TIMIT and TORGO adapted model. Table 4 presents the WERs in recognising the dysarthric speech of Nemours.

**Table 4:** WERs in recognising dysarthric speech using the adapted models of TIMIT and TORGO.

Experiment	Adapted Models			
	TIMIT		TORGO	
	MLLR	CMLLR	MLLR	CMLLR
MILD-A	9.23%	8.74%	10.65%	9.84%
MILD-B	13.95%	13.05%	15.54%	14.12%
MILD-C	11.06%	9.87%	11.98%	10.96%
MOD-A	30.17%	27.28%	33.98%	30.14%
MOD-B	50.24%	44.62%	44.03%	39.27%
MOD-C	42.62%	39.38%	37.94%	34.46%
SEV-A	77.82%	67.14%	67.92%	59.96%
SEV-B	67.68%	58.43%	58.42%	51.41%
SEV-C	71.44%	61.56%	62.95%	55.36%

**Figure 1:**
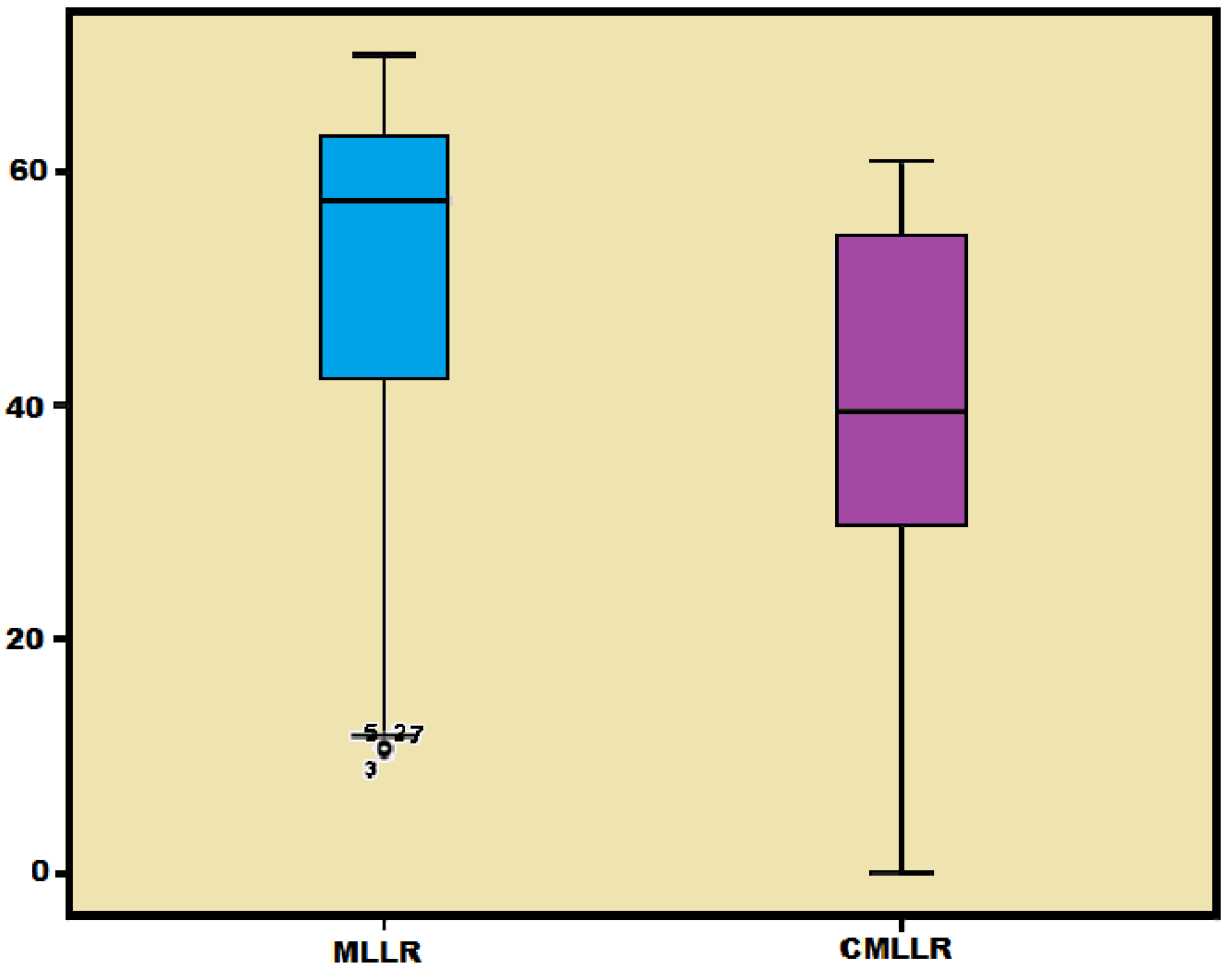
The box plot for the WER arising from the use of TIMIT adapted models.

**Figure 2:**
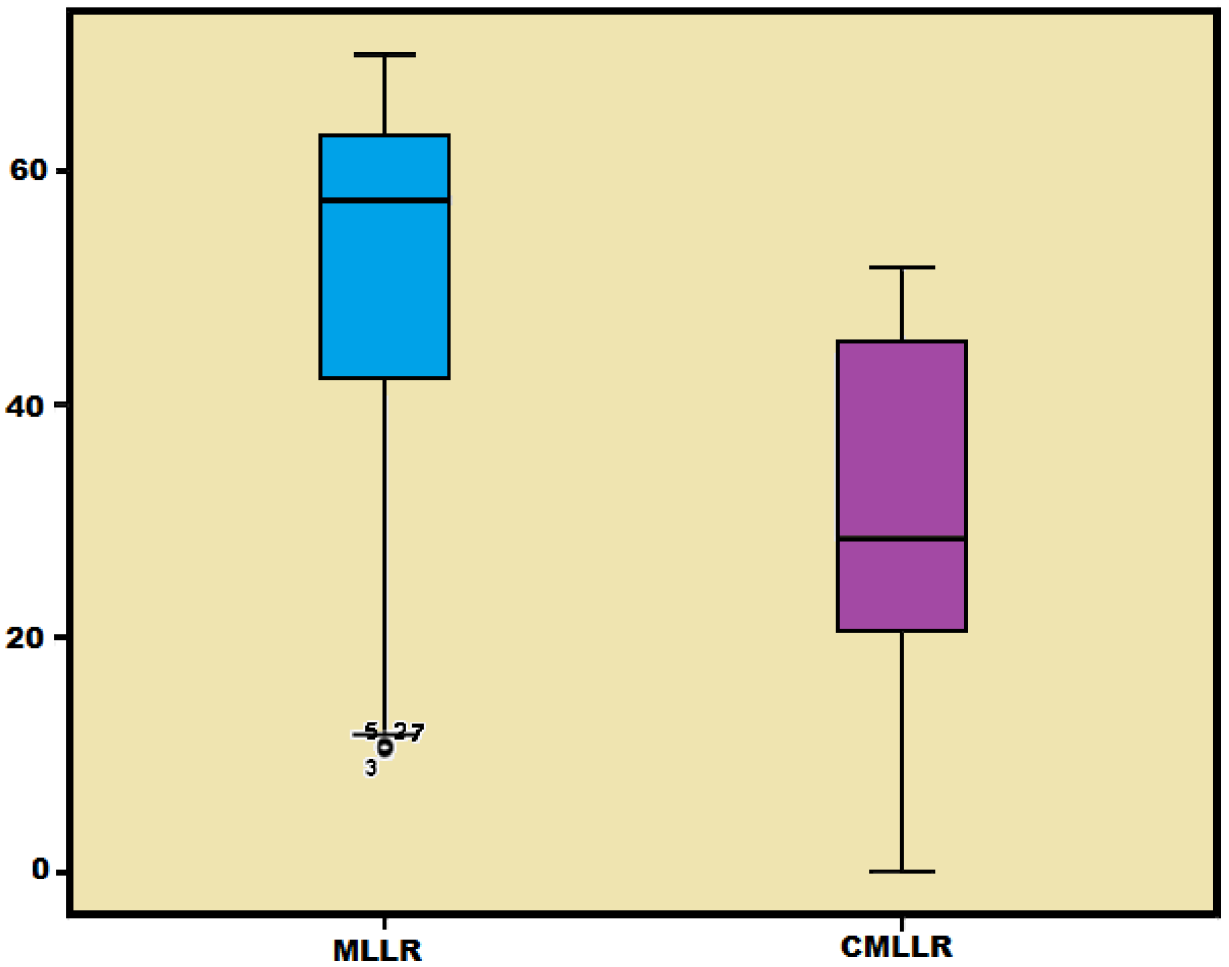
The box plot for the WER arising from the use of TORGO adapted models.

For the TIMIT and TORGO databases, we found that there is a significant difference in WER for MLLR and CMLLR techniques at p<0.05 (degree of freedom of 193 for TIMIT and 174 for TORGO. The F value is 4.759 for TIMIT and 6.880 for TORGO). Figures 1 and 2 show the box plot for the WER arising from the use of TIMIT and TORGO adapted models. For TIMIT based SI model as shown by Figure 1, the box plot of MLLR is relatively short as compared with that of CMLLR, which indicates that the WER of MLLR is significantly closer for all levels of severity. The box plot also shows that there is significant difference in WER between MLLR and CMLLR techniques. Similar results are also found for the TORGO-based SI model as shown in Figure 2. This shows that the adaptation technique plays an important role in recognition of dysarthric speech.

For each severity level, the results of the ANOVA test are presented in Table 5. Based on Table 5, it can be seen that the WER of the mildly and severe dysarthric speech is significantly different for the MLLR (denominator) and CMLLR (numerator) techniques for both the TIMIT and TORGO adapted models. However, we found no significant difference in mean for the moderately dysarthric speech.

**Table 5:** Results of the ANOVA test on WER for each severity level using the adapted models of TIMIT and TORGO.

Severity	Adapted Models	
	TIMIT	TORGO
Mild	significant difference in mean at p<0.05 (df = 17, F = 0.00, p = 0.00)	significant difference in mean at p<0.05 (df = 21, F = 3.876, p = 0.00)
Moderate	no significant difference in mean at p<0.05 (df = 65, F = 1.307, p = 0.300)	no significant difference in mean at p<0.05 (df = 63, F = 0.736, p = 0.787)
Severe	significant difference in mean at p<0.05 (df = 109, F = 2.080, p = 0.032)	significant difference in mean at p<0.05 (df = 88, F = 98.384, p = 0.00)

**Figure 3:**
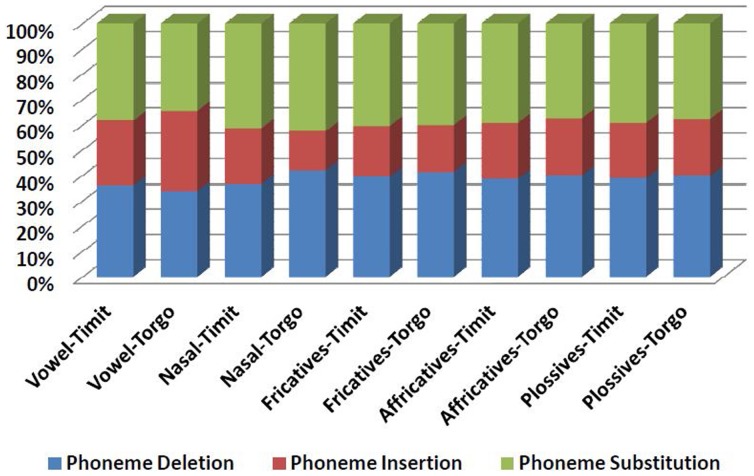
WER analysis in terms of phoneme deletion, insertion and substitution.

Our further analysis of WERs shows that phoneme substitution contributes to the highest WER in recognising the dysarthric speech for all experiments. Figure 3 shows the WER analysis in terms of phoneme deletion, insertion and substitution.

For phoneme insertion, the adapted model of TORGO shows a higher WER for the mildly and the moderately impaired speech, while the phoneme insertion is greater for the TIMIT, especially the severely impaired dysarthric speech. Plosives show the highest WER arising from phoneme insertion for all severity levels, followed by affricatives and fricatives. Nasals exhibit the lowest effect of phoneme insertion for both the TIMIT and the TORGO-adapted speech acoustic models.

For phoneme substitution, TORGO shows higher WER compared with TIMIT for the mildly dysarthric speech, whereas the phoneme substitution for the moderately and severely impaired speech is higher for TIMIT compared with TORGO. We found that the effect of phoneme substitution was the highest for plosives at all severity levels, followed by affricatives and fricatives.

For the WERs caused by phoneme deletion, the TIMIT adapted model shows greater WER for the severely impaired speech. On the other hand, the TORGO speech acoustic model has a higher phoneme deletion for the mildly and moderately impaired speech. The effect of phoneme deletion is the highest for plosives.

Table 6 shows the results for the ANOVA test to determine any significant difference in the mean of WER for each type of phoneme, particularly vowels, nasals and plosives for different adaptation models of TIMIT (denominator) and TORGO (numerator). For phoneme deletion, we did not find any significant difference in the mean of WER for vowels (mild, moderate and severe dysarthric speech), nasals (severe dysarthric speech), plosives (mild, moderate and severe dysarthric speech), and others (moderate and severe dysarthric speech) at p<0.05.

**Table 6:** The results of the ANOVA test to determine any significant difference in the mean of WER for each type of phoneme, particularly vowels, nasals and plosives for different adaptation models (TIMIT and TORGO).

Types	Severity	Phoneme type	ANOVA	df	F	p
Phoneme deletion	Mild	vowel	no significant difference	21	0.731	0.676
		nasal	significant difference	4	0.000	0.000
		plosives	no significant difference	21	0.816	0.648
		others	significant difference	4	0.000	0.000
	Moderate	vowel	no significant difference	39	0.651	0.781
		nasal	significant difference	9	0.000	0.000
		plosives	no significant difference	39	0.506	0.925
		others	no significant difference	15	1.358	0.450
	Severe	vowel	no significant difference	54	0.774	0.689
		nasal	no significant difference	16	3.500	0.399
		plosives	no significant difference	54	0.645	0.869
		others	no significant difference	26	0.680	0.763
Phoneme Insertion	Mild	vowel	no significant difference	16	0.943	0.308
		nasal	significant difference	2	0.000	0.000
		plosives	significant difference	48	8.569	0.000
		others	significant difference	20	20.175	0.000
	Moderate	vowel	no significant difference	31	0.871	0.603
		nasal	no significant difference	8	1.761	0.524
		plosives	significant difference	48	5.770	0.000
		others	no significant difference	20	6.301	0.070
	Severe	vowel	no significant difference	41	0.797	0.701
		nasal	no significant difference	9	0.668	0.713
		plosives	no significant difference	48	0.738	0.775
		others	no significant difference	20	0.519	0.855
Phoneme Substitution	Mild	vowel	significant difference	90	30.777	0.000
		nasal	significant difference	20	7.322	0.001
		plosives	significant difference	90	30.600	0.000
		others	significant difference	32	9.704	0.000
	Moderate	vowel	significant difference	90	11.256	0.000
		nasal	significant difference	20	5.150	0.014
		plosives	significant difference	90	8.340	0.000
		others	significant difference	32	8.443	0.000
	Severe	vowel	significant difference	90	3.383	0.000
		nasal	no significant difference	20	0.590	0.800
		plosives	significant difference	90	9.721	0.000
		others	no significant difference	32	0.428	0.952

For phoneme insertion, there is no significant difference in the mean of WER for vowels (mild, moderate and severe dysarthric speech), nasals (moderate and severe dysarthric speech), plosives (severe dysarthric speech), and others (moderate and severe dysarthric speeches). For phoneme substitution, we found that there is no significant difference in the mean WER for nasals (severe dysarthric speech), and others (severe dysarthric speech) at p<0.05.

Based on the ANOVA test, we found that the WER of TIMIT and TORGO did not show any significant difference in phoneme deletion and insertion, particularly for vowels and plosives at most of the severity levels. However, we did find significant difference in WER for phoneme substitution at all severity levels.

## Discussion

In this research, we have determined the performance of the ASR system in recognising impaired speech; the target model was adapted using the source model of both the unimpaired speech of TIMIT and the impaired speech of TORGO. The WERs of the two source models are different, with TORGO being better for recognising severe dysarthric speech while TIMIT is better for recognising mild dysarthric speech.

The adapted model built using CMLLR performed better than the model built using MLLR for all three levels of severity (mild, moderate and severe). This is because the CMLLR technique is used to extract features that are more specifically focused on the speaker-related speech properties rather than the standard spectral envelops [31]. This is supported by the ANOVA results that show a significant difference in the mean of WER for mild and severe dysarthric speech. However, we did not find any significant difference in variance of WERs for the moderate test data. The similarity in the ANOVA results reveals that the recognition of dysarthric speech depends more on the adaptation techniques rather than the type of adaptation source model.

In recognising the severely impaired dysarthric speech, the performance of the TORGO SI model is better than that of TIMIT although the former has a relatively smaller database. The better performance of the TORGO SI model is because the more severely impaired dysarthric speech has properties that are clearly different from those of the unimpaired speech, which are difficult to be integrated with the severely impaired dysarthric speech.

The results of the WER analysis show that the major factor causing the recognition error of dysarthric speech is phoneme substitution. Based on the ANOVA test, we found that the mean of WER of phoneme substitution is significantly different for the TIMIT and TORGO source models, especially for mildly and moderately dysarthric speech. From this test, we found that phoneme substitution is influenced by the level of severity of dysarthric speech.

The recognition error of consonants is much higher than that of vowels for all experiments. Among consonants, plosives have the highest occurrence of WERs in terms of phoneme insertion, substitution and deletion. The next highest WERs for consonants are affricatives and fricatives. The high WERs for these types of consonants are attributed to the speech properties of dysarthric speakers, especially imprecise articulation of consonants, prolonged phoneme and loudness decay.

## Conclusion

The biggest setback to the development of an ASR system for impaired speech is the small size of speech that can be acquired from a speaker with speech impairment. As such, it is vital for developers of ASR systems of impaired speech to seek alternative means for such development. Although the acoustic characteristics for unimpaired and impaired speech are indeed very different, the acoustic model of the former can be used as a source model for adapting the targeted impaired speech. The performance of the unimpaired speech acoustic model can be further improved using an effective adaptation technique. In this research, it was found that the CMLLR technique performs better than MLLR when using an unimpaired speech model.

The performance of the ASR system in recognising the speech of each speaker in the Nemours database is different, indicating a significant intra-speaker variation among dysarthric speakers. However, intra-speaker variability cannot be determined in this research due to the very small number of speakers in the Nemours database. Hence, intra-speaker acoustic variability warrants consideration in future work.
