# Correction: Severity-Based Adaptation with Limited Data for ASR to Aid Dysarthric Speakers

**DOI:** 10.1371/journal.pone.0097665

**Published:** 2014-05-08

**Authors:** The PLOS ONE Staff

In the Introduction under the subheading “B. Adaptation of Dysarthric speech”, Equation 1 is incorrect. The correct version of Equation 1 is:
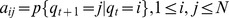
(1)

